# Effect of Off-Season Iron Supplementation on Aerobic Capacity of Female Handball Player: A Randomized, Double-Blinded, Placebo-Controlled Study

**DOI:** 10.1016/j.cdnut.2024.103767

**Published:** 2024-05-08

**Authors:** Ghazal Safa, Mohammad Hemmatinafar, Javad Nemati, Babak Imanian, Katsuhiko Suzuki

**Affiliations:** 1 Department of Sport Science, Faculty of Education and Psychology, Shiraz University, Shiraz, Iran; 2 Faculty of Sport Sciences, Waseda University, Tokorozawa, Japan

**Keywords:** aerobic power, handball players, iron supplementation, ventilation capacity, female athletes

## Abstract

**Background:**

Iron supplementation, especially in female athletes, is 1 of the influential factors in aerobic capacity, and its deficiency can lead to significant problems related to reduced aerobic capacity.

**Objectives:**

This study aimed to investigate the effect of 3 wk of iron supplementation on the aerobic capacity of female handball players.

**Methods:**

In this randomized**,** double-blinded, and placebo control trial, 14 elite handball players (age: 21.6 ± 5.68 y; height: 169.5 ± 4.9 cm; weight: 62.2 ± 9.25 kg; body mass index (in kg/m^2^): 21.5 ± 2.9) randomly divided into 2 supplement groups (receiving a 100 mg/d of poly-maltose tri hydroxide iron complex in the form of tablets) and the placebo group (receiving a tablet containing 100 mg/d starch which is the same color and shape as iron tablets). The supplementation protocol was performed for 3 wk during the off-season. Maximal oxygen consumption (VO_2max_), amounts of carbon dioxide at the first ventilatory threshold, amounts of carbon dioxide at the second ventilatory threshold, time to exhaustion (TTE), pulmonary ventilation (VE), ventilatory equivalents for oxygen, amounts of oxygen at the first ventilatory threshold, amounts of oxygen at the second ventilatory threshold, time to reach first ventilatory threshold, end-tidal partial pressure of oxygen at the first ventilatory threshold, end-tidal partial pressure of carbon dioxide at the first ventilatory threshold and ventilatory equivalents for carbon dioxide were measured using the Bruce test and gas analyzer in 2 pretest and posttest stages.

**Results:**

There were significant improvements in oxygen at the first ventilatory threshold, time to reach first ventilatory threshold, and end-tidal partial pressure of carbon dioxide at the first ventilatory threshold and a significant decrease in end-tidal partial pressure of oxygen at the first ventilatory threshold (*P* < 0.05). Also, no significant changes were found in VO_2max_, carbon dioxide at the first ventilatory threshold, carbon dioxide at the second ventilatory threshold, oxygen at the second ventilatory threshold, TTE, VE, ventilatory equivalents for oxygen, and ventilatory equivalents for carbon dioxide after 3 wk of iron supplementation (*P* > 0.05).

**Conclusions:**

The study found that 3 wk of off-season iron supplementation positively impacted female handball players’ aerobic capacity; however, it did not significantly improve their VO_2max_.

## Introduction

Modern handball is a team sport originating in Scandinavia in the early 19th century and was introduced as an Olympic sport in 1972 with the males’ discipline and 4 y later at the Olympic Games in Montreal with the females’ discipline. It is considered 1 of Europe’s most popular team sports, played by over 19 million worldwide [1]. Common factors such as nutrition, illness, and injury, as well as external influences and environmental conditions, can significantly influence sporting performance in handball players [2]. Therefore, adjusting handball athletes’ nutritional intake to meet their needs to cope with training and competition is becoming increasingly important [[3], [4], [5]]. However, knowledge of specific nutritional strategies during training and competition seems to need to be clarified among handball players. Similarly, energy, macronutrient, and especially micronutrient requirements such as iron need to be clearly understood according to the physical demands, locomotor power production, and exercise-induced anemia in handball athletes [6].

Anemia caused by sports activities has been repeatedly reported in various studies, and it seems that the presence of athletes in heavy competitive sports activities reduces the levels of iron, ferritin, and red blood cells (RBCs) [7,8]. Anemia has been reported, especially in intense endurance sports [9]. Among the main reasons for the reduction of blood iron and ferritin concentrations in professional athletes is the damage to RBCs in leg vessels due to hitting the ground and the damage to RBCs in torn capillaries in the digestive tract during intense activities [9,10]. Therefore, in extreme endurance sports, the athlete may experience a decrease in hemoglobin (Hb) concentration, which directly affects respiratory and cardiovascular function. In this context, it has been determined that the concentrations of iron, RBCs, and ferritin of Egyptian professional female runners have an inverse relationship with the distance they travel [11].

On the contrary, the gender difference in female athletes multiplies the role of iron in sports performance. A study conducted on 40 female mountaineers revealed that intermittent exercise with 100 mg iron increased the level and storage of iron, prevented the occurrence of anemia, and delayed fatigue by improving oxygenation [12]. Moreover, Hosseini et al. [13] (2019), on a group of 32 females, showed that 8 wk of aerobic exercise and the combined consumption of 100 mg iron and 500 mg vitamin C improved Hb, Hct, RBCs, ferritin, and transferrin (TRF). In addition, Córdova et al. [14] (2019), in a study conducted on 18 professional cyclists, showed that 4 wk of 80 mg/d iron supplementation improved iron, ferritin, Hb, and hematocrit (Hct) indices and muscle recovery. On the contrary, Ayosu et al. [15] (2018), in a study on 22 professional female volleyball players, showed that although 11 wk of iron supplementation increased blood iron concentrations, stopping consumption during the 18 wk of competition season caused a significant decrease in iron concentrations in professional female volleyball players. According to these results, it can be predicted that increasing iron concentrations and optimal changes in hematological indicators can improve aerobic capacity and sports performance in athletes, especially female athletes. Studies have investigated the effects of iron supplementation on changes in aerobic power and have found that the effects do not occur directly. Instead, they occur as a result of hematological changes and related adaptations [16]. In a 2013 study conducted by Ramezani and Mohammadioun [17], 37 female students aged 14–17 with iron deficiency disorder and healthy controls were given 100 mg ferrous sulfate daily for 45 d. The results showed that supplements helped people with iron deficiency reach normal serum iron (Fe) concentrations. However, their aerobic capacity was not significantly different from the control group’s [17]. Also, it has been established that active females taking 50 mg/d of iron supplements for 8 wk have increased iron concentrations and decreased lactate concentrations during sub-maximal activity [16]. Researchers Omidali and Hamzeloo [18] observed that boys aged 15–18 who consumed 100 mg iron supplements daily and 6 wk of periodic training showed increased aerobic capacity and specific blood indices. Female athletes are at a higher risk of iron deficiency due to monthly menstruation, making it necessary for them to take additional iron supplements [19]. Athletes with optimal blood iron concentrations perform better in heavy sports activities and can delay fatigue [13].

Therefore, iron is a supplement that significantly affects the performance of sports activities and can delay muscle fatigue [20]. Iron plays a vital role in oxygen transport to tissues through Hb, oxygen storage in muscles through myoglobin and multiple processes involved in the oxidative regeneration of ATP. The optimal amount of iron is 1 of the most influential factors in sports performance capacity, especially in female athletes, and is directly related to their health [21]. Iron can also increase maximal oxygen consumption (VO_2max_) and decrease blood lactate concentration [22]. Considering the importance of oxygen consumption in athletes of various disciplines and the fact that iron increases the amount of oxygen uptake by ∼65%, its deficiency can lead to significant problems related to reducing an athlete’s aerobic capacity [23]. Some researchers believe exercise training increases the daily iron requirement [24]. For example, the need for iron increases during sports activities due to its loss through sweating. Also, the destruction of RBCs due to mechanical blows causes the loss of blood Hb through urine. Finally, due to the critical role of iron in the transport and consumption of oxygen, the capacity to perform sports activities decreases [25]. Taking iron supplements increases iron concentrations, ferritin, RBCs, Hb, Hct, and blood transfer [20].

Based on the information provided, more study is necessary on the impact of iron supplementation on athletes’ performance, particularly in the case of female handball players. As iron plays a crucial role in oxygen supply and aerobic capacity, this study aimed to investigate the effect of 3 wk of iron supplementation on the aerobic capacity of female handball players.

## Methods

### Participants

Fourteen elite female handball players were recruited from the Shiraz handball team. The inclusion criteria were age 18–25 y, 2 y of activity in the Premier League, and perfect physical health. The exclusion criteria were refusing to participate in the supplement protocol, contracting diseases such as COVID-19 or other diseases during research, iron deficiency, and using medicine or dietary supplements [26]. Also, the present study was approved by the Ethics Committee of Shiraz University, Shiraz, Iran (IR.US.REC.1401.023) and was performed by the Declaration of Helsinki. Additionally, the study was registered with the Iranian Registry Clinical Trial and assigned the code IRCT20230816059165N1. All participants provided written informed consent before enrolling in the study. Moreover, all participants were members of the same training camp, and their training regime was the same under the supervision of trainers.

### Study design

**FIGURE 1:**
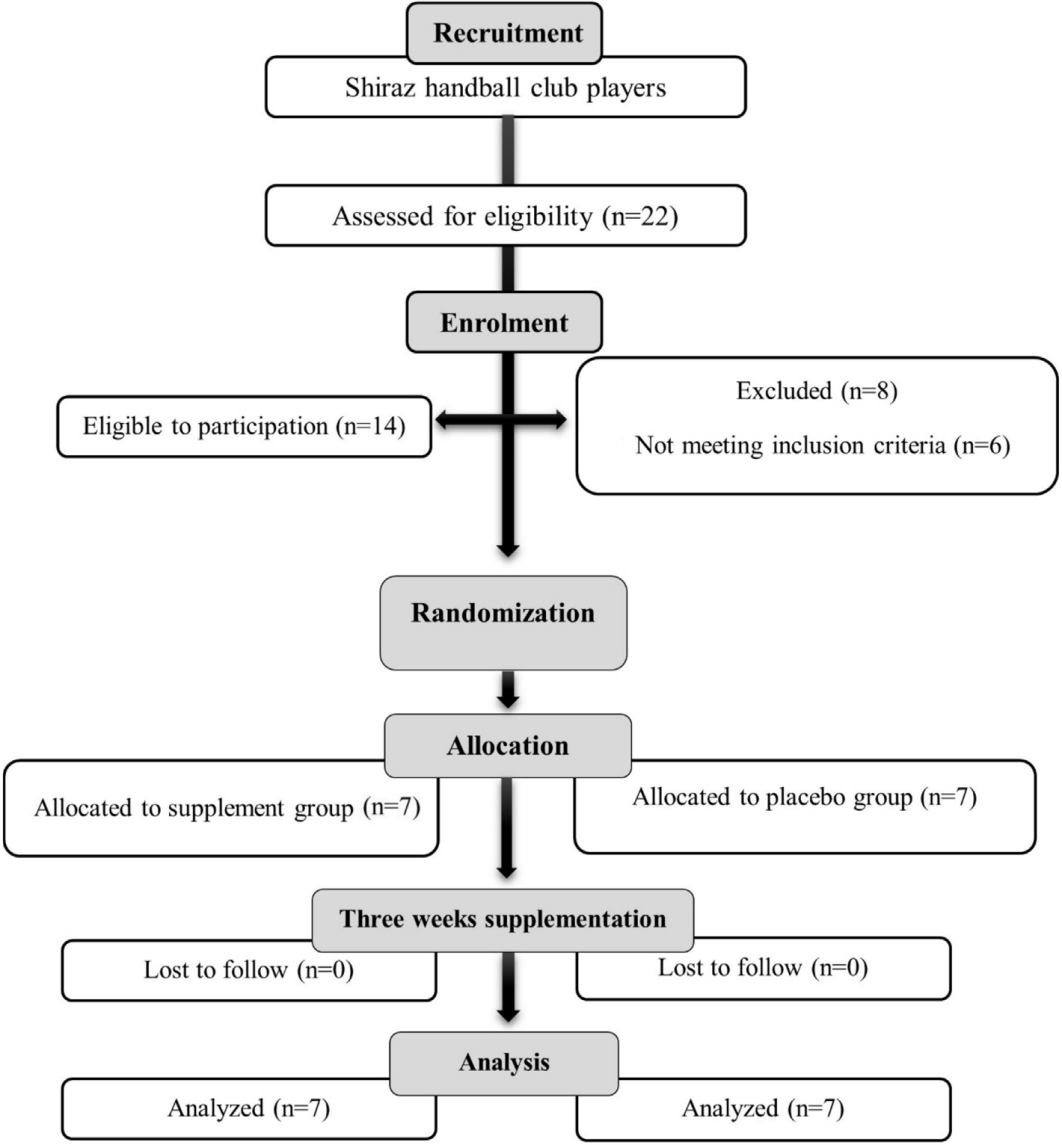
Flowchart illustrating the different phases of the research and study selection.

The present research was a randomized, pre and posttest, double-blinded, and placebo control study. After checking the general health of these players by a general practitioner, completing the physical activity readiness questionnaire test, and the written consent form for participation in the research (Including full descriptions of the implementation method, benefits, risks, and possible complications), participants were randomly divided into supplement or placebo groups. Using block randomization, eligible participants were randomly assigned in a 1:1 ratio to either the supplement or placebo group [27]. Participants were instructed to avoid consuming caffeinated sources and perform vigorous exercise ≥24 h before the experiment to control for any acute and side effects on the measured variables [28]. The randomization allocation table was created by a study team member blinded to assessments; allocation numbers were then placed in sealed envelopes (Figure 1). It should be noted that all participants were fed the same breakfast containing 250 kcal (45 g carbohydrates, 9 g protein, and 5 g fat) 1 h and 30 min before the test sessions [26]. Additionally, the pretest and posttest were performed at the same time of day (8:30–13:00). During the trials, participants were allowed to drink water independently.

### Supplementation protocol

In the current study, according to the amount of daily iron consumption in previous studies, which varied between 30 and 160 mg/d [[12], [14]], the amount of iron consumed by the experimental group was 100 mg/d (1 tablet of 100 mg iron hydroxide complex maltose along with a 150 mL glass of water in 1 meal), and the placebo group received 100 mg placebo daily (a starch capsule that is the same color and shape as iron tablets and is completely ineffective). The supplementation protocol was performed for 3 wk in the off-season. Also, all the participants were taught about the possible side effects of taking iron supplements. We asked them to stop taking the supplement if they had any symptoms, and they informed us that none of the participants suffered these side effects. It should be noted that all participants were members of the same training camp, and their training regime was the same under the supervision of trainers.

### Measurements

The participants’ anthropometric data (age, height, weight, and BMI) were first measured in the pretest session (Table 1). After warming up, based on the study by Tomescu et al. [29], the subjects did the Bruce test on the treadmill (h/p/cosmos, Sports & Medical GMBH). Also, their aerobic capacity and endurance performance indicators such as VO_2max_, amounts of carbon dioxide at the first ventilatory threshold (VT) (VT_1_ CO_2_), amounts of carbon dioxide at the second VT (VT_2_ CO_2_), time to exhaustion (TTE), pulmonary ventilation (VE), ventilatory equivalents for oxygen (EQO_2_), amounts of oxygen at the first VT (VT_1_ O_2_), amounts of oxygen at the second VT (VT_2_ O_2_), time to reach first VT (Time VT_1_), end-tidal partial pressure of VT_1_ O_2_ (PETO_2_ VT_1_), end-tidal partial pressure of VT_1_ CO_2_ (PETCO_2_ VT_1_) and ventilatory equivalents for carbon dioxide (EQCO_2_) measured by a respiratory gas analyzer device (MetaLyzer 3B; Cortex). After 3 wk of supplementation, the subjects participated in the posttest session. All the tests were performed the same as the pretest, and the relevant data were recorded.

**TABLE 1:** Iron status report of participants

Variable	Serum values (*n* = 14)
Fe (μmol/L)	24.00 ± 3.81
FER (μg/L)	119.00 ± 12.53
TSI (%)	27.00 ± 6.19
TRF (μg/dl)	341.00 ± 16.31
Hb (g·L^−1^)	19.00 ± 3.49
Hct (%)	39.00 ± 1.19

Abbreviations: Fe, serum iron; FER, serum ferritin; g, gram; Hb, hemoglobin; Hct, hematocrit; L, liter; TRF, transferrin; TSI, transferrin saturation index; *μ*, micro.

### Kinetic oxygen conception parameters

VT: The VT was visually determined using the modified V-slope method described by Sue et al. [30], which is a modification of the method described by Beaver et al. [31]. The ventilatory equivalent method (the point at which VE/VO_2_ begins to rise without an increase in VE/VCO_2_) and end-tidal methods [partial pressure of end-tidal oxygen tension (PETO_2_) start to grow without a decrease in partial pressure of end-tidal carbon dioxide (PETCO_2_)] was used as a complement [32]. The current study measured and reported the oxygen consumption equivalent at VT1 (VT_1_ O_2_).

### Iron status report

According to the participants’ medical reports, the values of Fe, serum ferritin, TRF saturation index, TRF, Hb, and Hct were checked. The results showed that all 14 participants in the study had adequate iron stores (Fe: 11–29 *μ*mol/L, serum ferritin >100 *μ*g/L, TRF saturation index >20%, TRF: 300–360 *μ*g/L, Hb > 12 g·L^−1^, Hct: 36–48%) [15,33] (Table 1).

### Statistical analysis

Descriptive statistical indicators such as mean and SD were used in this study. The normal distribution of data and homogeneity of variance were checked using Shapiro-Wilk and Leven tests. An independent-sample t-test was used for between-group analysis, and a paired-sample t-test was used for within-group analysis. The significance level was set at *P* ≤ 0.05. All analyses were done using SPSS software version 26 (IBM-SPSS Inc), and GraphPad Prism software (GraphPad Software Inc.) was also used to design the graphs.

## Results

**FIGURE 2:**
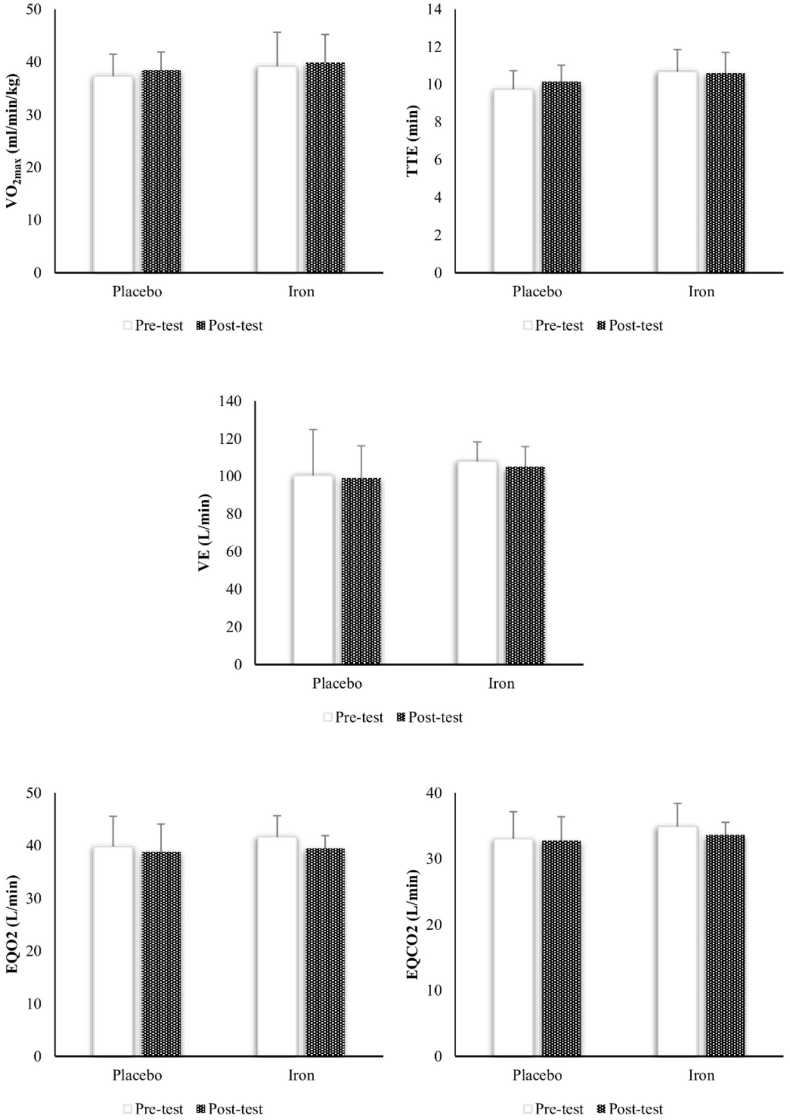
Pre and posttest changes of study outcomes in study groups. EQCO_2_, ventilatory equivalents for carbon dioxide; EQO_2_, ventilatory equivalents for oxygen; TTE, time to exhaustion; VE, pulmonary ventilation; VO_2max_, maximal oxygen consumption.

**FIGURE 3:**
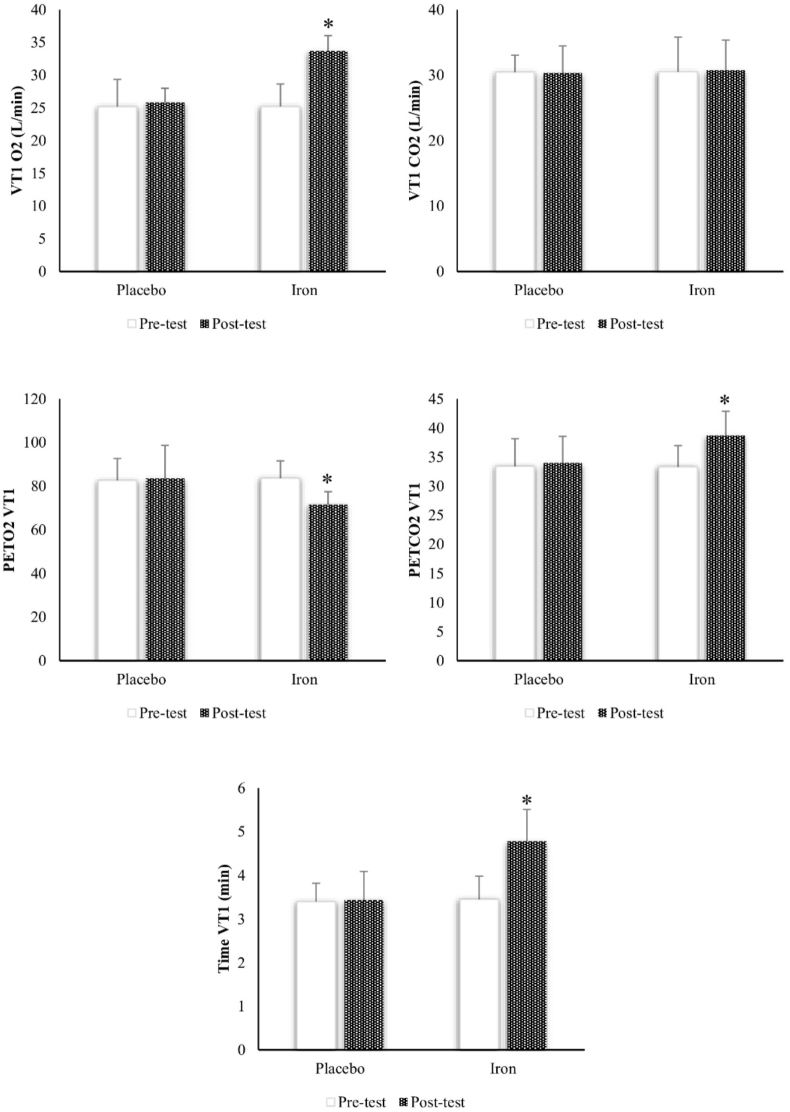
Pre and posttest changes of study outcomes in study groups. PETCO_2_ VT_1_, end-tidal partial pressure of carbon dioxide at the first ventilatory threshold; PETO_2_ VT_1_, end-tidal partial pressure of oxygen at the first ventilatory threshold; Time VT_1_, time to reach first ventilatory threshold; VT, ventilatory threshold; VT1 CO_2_, carbon dioxide at the first ventilatory threshold; VT1 O_2_, oxygen at the first ventilatory threshold. ∗: Significant difference compared to the Placebo group.

**FIGURE 4:**
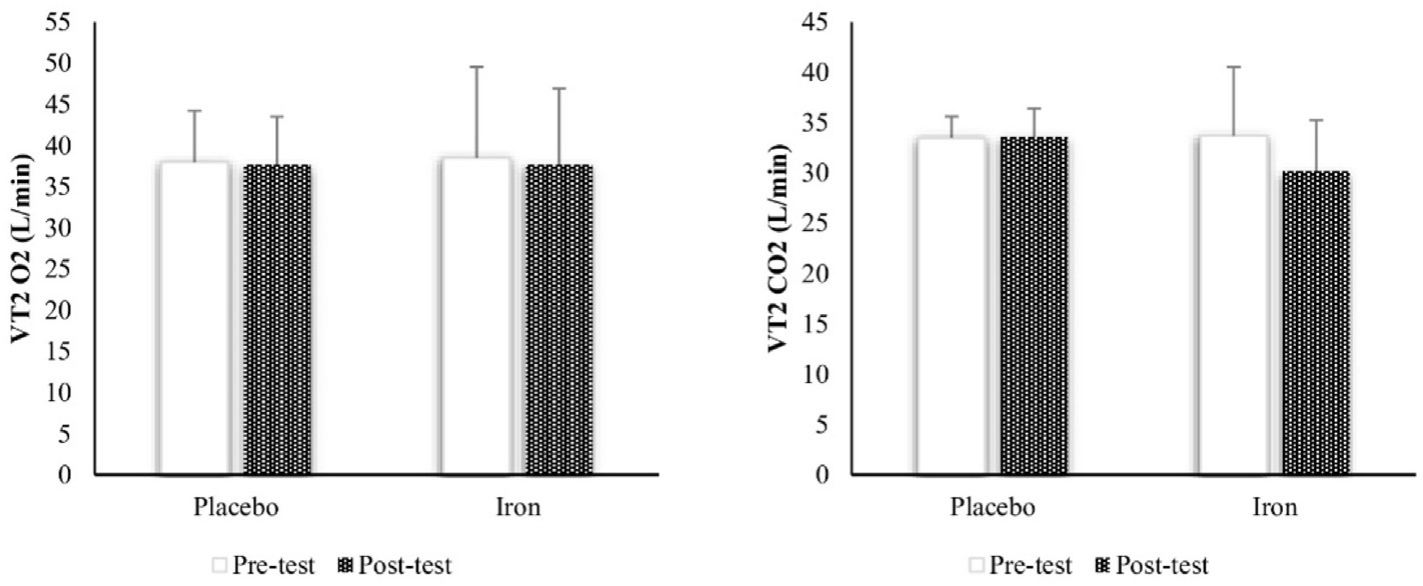
Pre and posttest changes of study outcomes in study groups. VT_2_ CO_2_, carbon dioxide at the second ventilatory threshold; VT_2_ O_2_, oxygen at the second ventilatory threshold.

Table 2 presents the study groups’ baseline descriptive characteristics. There were no significant differences in age, height, weight, and BMI between the groups at baseline (*P* ≥ 0.05). The analysis of the paired-sample t-test did not show any significant differences between the placebo and supplement groups for all study outcomes (*P* ≥ 0.05), as shown in FIGURE 2, FIGURE 3, and **4**.

**TABLE 2:** Descriptive characteristics of study groups at baseline

Variable	Placebo	Iron	*P* value
Age (y)	20.28 ± 5.82	21.85 ± 5.52	0.614
Height (cm)	168.00 ± 5.68	171.14 ± 4.25	0.265
Weight (kg)	61.71 ± 7.13	62.71 ± 11.38	0.847
BMI (kg/m^2^)	21.85 ± 2.26	21.28 ± 3.54	0.726

The result of between-group comparisons by independent-sample t-test analysis indicated that there were significant improvements in VT_1_ O_2_ (T = 3.701, *P* = 0.031), Time VT_1_ (T = 7.293, *P* = 0.001) and PETCO_2_ VT_1_ (T = 6.170, *P* = 0.001). A significant decrease was also observed in PETO_2_ VT_1_ (T = 4.655, *P* = 0.042). Nevertheless, there were no significant differences in VO_2max_ (T = 0.318, *P* = 0.756), VT_1_ CO_2_ (T = 0.381, *P* = 0.250), VT_2_ CO_2_ (T = 2.440, *P* = 0.065), VT_2_ O_2_ (T = 1.670, *P* = 0.089), TTE (T = 1.609, *P* = 0.134), VE (T = 0.266, *P* = 0.795), EQO_2_ (T = 0.691, *P* = 0.503) and EQCO_2_ (T = 0.731, *P* = 0.479) (FIGURE 3, FIGURE 4).

## Discussion

The purpose of this study was to determine the effect of 3 wk of iron supplementation on VO_2max_, VT_1_ CO_2_, VT_2_ CO_2_, TTE, VE, EQO_2_, VT_1_ O_2_, VT_2_ O_2_, Time VT_1_, PETO_2_ VT_1_, PETCO_2_ VT_1,_ and EQCO_2_ in female handball players. The results indicated significant VT_1_ O_2_, time VT_1_, and PETCO_2_ VT_1_ improvements. Also, a significant decrease was observed in PETO_2_ VT_1_. However, no significant changes were found in VO_2max_, VT_1_ CO_2_, VT_2_ CO_2_, VT_2_ O_2_, TTE, VE, EQO_2_, and EQCO_2_ after 3 wk of iron supplementation in female handball players.

Iron metabolism in response to exercise is a highly disputed topic in exercise physiology with various perspectives. Iron is essential to Hb, transporting oxygen from the lungs to all body cells. It is also a vital part of enzyme systems involved in energy production and metabolism [34]. Optimal blood iron concentrations positively affect athlete performance in heavy sports activities and can delay fatigue [13]. Iron supplementation in females with ferritin <20 ng/mL showed a significant increase in TTE in the supplemented group [35]. This result is inconsistent with the findings of the present study. The most important reason is probably the difference in the concentrations of ferritin and iron status between the participants of the 2 studies. Pourmohak et al. [12] (2022), in a study on 40 female mountaineers, showed that interval training with the consumption of 100 mg iron increased the level and storage of iron and prevented the occurrence of anemia. Also, it delays fatigue by increasing the oxygen supply. Besides, it was shown that iron supplementation is associated with significantly improving muscle fatigue and resistance to fatigue during exercise in females [20]. On the contrary, a study investigated the effect of iron supplementation on the endurance capacity of female runners. It concluded that TTE did not significantly differ between the placebo and supplemented groups [36]. These results are in line with the findings of the current study. It has also been reported that an increase in VO_2max_ leads to a delay in fatigue and an increase in TTE [37]. On the contrary, examining the effect of iron supplementation on the aerobic capacity of 17–14-y-old female students with different levels of iron deficiency showed that although iron supplementation compensates for iron deficiency, its effect on aerobic power is insignificant [17]. This result is in line with the result of the present study. It was reported that if aerobic exercise by young girls is accompanied by iron supplementation, in addition to increasing the amount of iron, the storage of iron in the form of ferritin and TRF also increases and probably prevents anemia in them [38]. It was also stated that iron supplementation can increase hematological factors, i.e., Fe and ferritin, and positively affect aerobic capacity [39]. Another study investigated various aspects of iron supplement consumption in athletes. Finally, it showed that male and female athletes often obtain the recommended amount of iron of 10–15 mg/d through their diet [40]. It also showed that after iron supplementation, there was a significant increase in the VO_2max_, and the results of their study were inconsistent with the present study’s findings that iron supplementation cannot affect aerobic capacity (VO_2max_). On the contrary, in line with the present studies, several studies did not show a significant relationship between iron supplementation and improving aerobic capacity and endurance performance [13,19,20]. One of the main reasons that conflicted with the results of the present study may be the difference in duration and amount of supplementation. For example, in the study of Friedman et al. [41], the subjects underwent iron supplementation for 6 wk; however, in this study, the duration of the supplementation period was 3 wk.

No study was found on the effect of iron supplementation on EQO_2_ and EQCO_2_. In exercise physiology, oxygen ventilation equivalent is used to determine the fitness level of athletes, respiratory efficiency, and the noninvasive estimation of lactate threshold. A change in the amount of pulmonary ventilation per minute (VE/min) can cause fluctuations in EQO_2_ and EQCO_2_ [42]. In the current study, as there was no significant change in VE/min, it is evident that EQO_2_ and EQCO_2_ did not exhibit any change either. Considering the richness of cumin in iron (over 66 mg/100 mg), it showed that cumin supplementation on VE, EQO_2_, EQCO_2_, and aerobic capacity (VO_2max_) did not significantly affect student-athletes [43]. Rubeor et al. [44] (2018) conducted a review study and found that female athletes who suffer from iron deficiency can improve their physical performance by taking iron supplements. They also noted that iron supplementation significantly impacts individuals with ferritin concentrations below 20 IU/L [44]. Considering that the participants of the present study were not suffering from iron deficiency, 100 mg iron supplement consumption for 3 wk could not significantly affect the aerobic endurance capacity of female handball players, especially VO_2max_ and VE/min.

On the contrary, it stated that iron supplementation can increase hematological factors such as Fe and ferritin and positively affect aerobic capacity [39]. It showed that the effects of iron deficiency on physical performance and physical activities depend on the degree of iron deficiency [45]. In general, because it has been reported that there is conflicting evidence about the severity of iron deficiency due to exercise and its effect on the performance of female athletes, the reduction of iron in exercise is the result of some mechanisms during exercise, such as hemolysis, sweating, and gastrointestinal and intestinal bleeding [46]. As a result of such a reaction, the amount of cytokines and, as a result, the liver product of the hormone hepcidin increases, and the increase of hepcidin has a negative effect on iron transfer and absorption channels in the body [47]. Although females are the leading group at risk of moderate anemia, most studies do not show significant changes in relation to iron supplementation or the physical capacity of athletes with pre-existing iron deficiency [48]. Therefore, using iron supplements in athletes who have suffered from iron deficiency anemia improves the effective indicators of performing sports activities and thus improves their performance. It seems that using iron supplements by athletes who do not suffer from iron deficiency anemia will not increase sports performance [47]. Iron supplementation has been recommended by young female endurance and marathon runners, who are more prone to losing iron stores than other athletes [49]. Nevertheless, it has been shown that taking iron supplements does not improve an athlete’s performance. The theory suggests that iron availability during physical activity stimulates blood production, which helps maintain the concentration of the photoprotein factor during training [50]. The last step in producing heme involves the introduction of iron into protoporphyrin, which relies on the availability of iron as a primary material. Iron also governs and monitors the initial stage of heme production and the development of the 5-aminolevulinate factor. Therefore, there are no practical restrictions in the hematopoietic process with the presence of iron in athletes with natural iron reserves [39].

Based on the information presented above, it can be concluded that the consumption of 100 mg iron for 3 wk may not have the same effect on subjects who are not suffering from iron deficiency as compared to female athletes with iron deficiency, who have shown a significant increase in their aerobic capacity (VO_2max_) after taking iron supplements. However, taking iron supplements for 3 wk can still positively affect some factors related to endurance capacity in female handball players, even if they are not suffering from iron deficiency. It is also important to note that the study was conducted outside of the competition season. This means that the possibility of sports anemia, which is caused by intense exercise and can lead to a decrease in RBCs and Hb, was reduced due to the lower intensity of the exercises, more extended recovery periods, and reduced exercise-induced muscular damage. Finally, the study focused on the effect of iron supplementation on the aerobic capacity of handball players. Given the intermittent nature of handball, where players are not at maximum intensity throughout the entire match, even a relative improvement in some aerobic capacity factors can positively affect the player’s performance.

This study had some limitations that should be addressed in future research. Firstly, due to the COVID-19 pandemic, the Sports Medicine Federation of Iran prohibited blood and tissue sampling from athletes. This prevented us from measuring the iron concentrations of participants before and after the iron supplementation period. However, we reviewed the medical reports of the participants and confirmed that none of them were suffering from iron deficiency. Secondly, the intervention protocol was implemented in the off-season of athletes’ competitions due to COVID-19 quarantine restrictions. However, the competition season is when sports anemia is more likely to occur due to the higher intensity of activities, less time for recovery, and the compression of competitions and training. Furthermore, it is essential to note that the sample size used in this study was limited due to the nature of a complete handball team, which typically consists of 12–14 players. Therefore, future studies should incorporate the evaluation of these variables to enhance our understanding of the topic.

In conclusion, according to the results of the present study, 3 wk of 100 mg iron supplementation can be approximately effective on the aerobic capacity of female handball players, especially VT_1_ O_2_ and Time VT_1_. In addition, consuming 3 wk of 100 mg iron could significantly improve their PETCO_2_ VT_1_ and PETO_2_ VT_1_; however, it did not substantially improve their VO_2max_. Additionally, based on the findings of this study, female handball players may benefit from iron supplementation during the off-season period to maintain their aerobic performance.

### Author contributions

The authors’ responsibilities were as follows– GS, MH, JN, BI, KS: designed the study; GS, BI: were involved in data collection; GS, MH, BI: responsible for data entry, statistical analysis, and writing the manuscript for the current study; JN, KS: primary responsibility for the final content; All authors contributed to the interpretation of the findings; all authors: read and approved the final manuscript.

### Conflict of interest

The authors report no conflicts of interest.

### Funding

MH reports administrative support, article publishing charges, equipment, drugs, or supplies, and statistical analysis were provided by Shiraz University.

### Data availability

Data described in the manuscript, code book, and analytic code will be available upon reasonable request.
